# Influence of Land-Use Intensification on Vegetation C-Stocks in an Alpine Valley from 1865 to 2003

**DOI:** 10.1007/s10021-017-0120-5

**Published:** 2017-03-10

**Authors:** Maria Niedertscheider, Erich Tasser, Monika Patek, Johannes Rüdisser, Ulrike Tappeiner, Karl-Heinz Erb

**Affiliations:** 1 Institute of Social Ecology Vienna, Alpen-Adria Universitaet, Schottenfeldgasse 29, 1070 Vienna, Austria; 2 Institute for Alpine Environment, European Academy Bozen/Bolzano, Bolzano/Bozen, Italy; 3 Department of Botany and Biodiversity Research, Division of Conservation Biology, Vegetation and Landscape Ecology, University of Vienna, Vienna, Austria; 4 Institute of Ecology, University of Innsbruck, Innsbruck, Austria

**Keywords:** carbon stock, LTSER, HANPP, land-use intensity, forest management, socio-economic drivers, energy transition

## Abstract

The role of ecosystems as carbon (C) sinks or sources is intrinsically related to land-use intensity, which determines the land required for biomass production. Here, we systematically investigate the role of different land-use types including their land-use intensities on vegetation C-stocks (SC_act_) in the Stubai valley, located in the Austrian central Alps. After a period of high land-use impacts until 1954, indicated by massive C-depletion, land-use shifted to completely new courses. Polarization into high-intensity low-land areas and extensification at higher altitudes allowed for a tripling of SC_act_ until 2003. The most important land-use change was the intensification of the livestock sector accompanied by abandonment of extensive grasslands and reduced harvest pressure on forests after WWII. Market integration, abundance of fossil energy carriers, as well as structural change of the economy were important underlying socio-economic drivers of these trends. However, despite this remarkable SC_act_ increase, SC_act_ amounted to only 62% of the potential carbon stocks (SC_pot_) in 2003. Although conversion of forests to agriculture clearly contributed the lion’s share to this SC-gap, forest management explains roughly one quarter of the SC-difference. We found that time-lags between land-use shifts and the establishment of a new C-climax had fundamental repercussions on recent C-dynamics in the study region. Apparently, the land system is still net-accumulating C, although land-use changes have peaked decades earlier. Our findings are crucial for the understanding of C-dynamics, including the role of land management and time-lags in mountainous regions, which are regarded key areas for terrestrial C-sequestration.

**Electronic supplementary material:**

The online version of this article (doi:10.1007/s10021-017-0120-5) contains supplementary material, which is available to authorized users.

## Introduction

Carbon (C) sequestration in vegetation and soils represents a promising strategy to offset C emissions from fossil fuel combustion (IGBP Terrestrial Carbon Working Group 1998; Canadell and others 2007; Houghton 2014) particularly in a short-term perspective (Mackey and others 2013). Special attention has been paid to mountainous areas in Europe and North America, where land-use processes in the past have resulted in a marked regrowth of natural vegetation (MacDonald and others 2000; Piussi and Pettenella 2000; Gingrich and others 2007; Zimmermann and others 2010; Kuemmerle and others 2011; Niedertscheider and Erb 2014; Gingrich and others 2015; Vigl and others 2016), often forests, that store high amounts of C (Meyfroidt and Lambin 2011; Locatelli and others 2016). Particularly, the substitution of fuel-wood with modern energy carriers, biomass imports and agricultural intensification have been identified as pivotal drivers of these trends in the twentieth century (Mather 1992; Mather and Needle 1998; Rudel and others 2005, 2010), determining the way land is managed for biomass production and the level of C-stocks (SC_act_).

C-accumulation witnessed in recent years mainly occurred on areas that were forests before being converted to agriculture centuries and millennia ago (Mather 1992; Grainger 1995; Mather and Needle 1998). By converting forests to agricultural land, societies have substantially reduced natural C-stocks (SC_pot_), mainly by replacing perennial plant species, such as trees, with annual crops, or grass-types (Erb 2004; Houghton and others 2012; Le Quéré and others 2015). Also forest management, often accompanied by a reduction in stand age and deadwood component and a change of species composition, contributed to SC-reductions (Schulze and others 2012; Fontana and others 2013; Erb and others 2016a). Hence, recent C-accumulation likely represents a compensation of past depletion. Quantifying the difference between C-stocks today (SC_act_) to the pre-agricultural system (SC_pot_) appears worthwhile for assessing the net-C debt of land conversion and land management (Gingrich and others 2007; Erb and others 2008).

Although effects of land cover change on SC_act_ have been systematically investigated before (Pongratz and others 2009; Houghton 2014; Fuchs and others 2015), many knowledge gaps relate to the role of land management and land-use intensity in particular (Erb and others 2013a, 2016b), due to several interrelated reasons. Firstly, traditional land system analyses tend to use land cover classification or plant functional types as units of analysis and thus fails to integrate the variety of C-densities of one and the same land cover type depending on the intensity of use (Tappeiner and others 2008; Erb 2012; Bloom and others 2016; Erb and others 2016a). Secondly, integrating SC_act_ with land-use intensity changes requires an enormously rich data basis, including areas and input–output intensities of all occurring land cover types in a region, which is often not available. Related to that, possible time-lags between land-use changes and when a new C-climax is established require time-series analysis to scrutinize legacy effects (Willis and Birks 2006; Liu and others 2007; Gaillard and others 2010; Singh and others 2013; Bürgi and others 2016). The long-term perspective also warrants a deeper understanding about the socio-economic framework conditions under which changes in land use allow for shifting from a C-source to a sink, or vice versa. Finally, quantifying the role of land-use intensity on SC-changes requires a management-independent reference system that allows to contrast actual, that is, human-managed, with natural C-states. In several studies, the concept of potential vegetation, that is, the hypothetical vegetation that would prevail without land use has proven useful for this purpose (Erb and others 2013a; Haberl and others 2014).

In this study, we aim at providing a comprehensive perspective on the role of land-use intensification for SC_act_ trajectories in mountainous regions, based on evidence from the municipality of Neustift in the Stubai valley (hereafter referred to as Neustift), Tyrol, Austrian Central Alps. The Stubai valley has been categorized as an archetypical “standard region”, with regard to specialization on livestock farming and a high tourism component (Tappeiner and others 2008). Neustift covers approximately 80% of the Stubai valley, spans an area of 250 km^2^ and ranges between 533 and roughly 3500 m a.s.l. For the Stubai valley, which has been an LTSER site since 1992 (Tappeiner and others 2013; Kerle and Tappeiner 2017), an extraordinarily rich data base of historical land use and forest SC_act_ exists (Tappeiner and others 2008; Tasser and others 2012; Patek 2013). We will use this database and develop it further to analyse the relation between land management and SC_act_ changes from 1865 to 2003 in a spatially explicit way.

We will apply the analytical framework of Human Appropriation of Net Primary Production (HANPP; (Haberl and others 2007, 2014 Erb and others 2009) as a tool to quantify and operationalize land-use intensity changes. HANPP represents an indicator framework for quantifying land-use dynamics by integrating the impacts of two processes on ecosystem energy flows: (a) harvest (HANPP_harv_) and (b) land-use-induced modifications of NPP, defined as the differences between potential NPP (NPP_pot_; NPP that would prevail in ecosystems without land use) and actual NPP. HANPP allows for separation of human from natural drivers of changes and has been used for land-use intensity studies before (Niedertscheider and others 2014, 2016; Niedertscheider and Erb 2014; Gingrich and others 2015). HANPP integrates the socio-economic with the ecological dimensions of land-use intensification (Erb and others 2013a): HANPP_harv_ serves as an indicator for output intensity, revealing the societal benefits of land use, while absolute HANPP levels indicate the overall impact of land use on ecosystem NPP flows and thus serve as an indicator for system-level intensity (Erb and others 2013a). Using Neustift as a case study and model region, we aim to answer the following research questions:

1. How did spatial patterns, trends and levels of SC_act_ develop over the past 140 years?
2. How has land-use intensity measured by HANPP changed for different land-use types?
3. What is the contribution of area changes versus intensity changes of different land-use types to SC- and NPP-dynamics?
4. What was the influence of socio-economic versus climatic dynamics on C-trajectories?
5. How do time-lags between land-use changes and new SC steady-states affect the status of SC_act_?

## Materials and Methods

Our analysis of land-use intensification, measured through HANPP, and its influence on SC-trajectories involved several steps. (1) We quantified above- and belowground vegetation SC_act_ in Neustift for the years 1865, 1954, 1973, 1988 and 2003. (2) We quantified above- and belowground NPP flows (NPP_act_) and (3) the amount of harvested NPP (HANPP_harv_) for the same time steps. (4) We contrasted these assessments with quantifications of potential NPP (NPP_pot_) and SC (SC_pot_) in vegetation and (5) calculated HANPP flows for all time steps. To analyse the role of socio-economic changes and climatic changes and to identify possible underlying drivers contributing to these trends, we (6) compiled a set of socio-economic and climatic variables for the considered time steps and study area. All six steps are described in more detail in the sections below, and Table 1 provides a summary of the data and main methods we used. Additional detailed information is available in the supplementary online material (SOM). For all calculations, we used the original aggregation level of 24 LULC-classes (SOM Table S1), which we then aggregated to seven classes for the representation and discussion of results.

**Table 1:** Data and Methods Used for the Empirical Work of This Study

LULC-type	Data used	Main methods
SC_act_		
Forests	Primary sources: Historic forest inventory maps (see Patek 2013)	Data used directly for 1954, 1976 and 2003; recalculation for years 1865 and 1988
Shrublands	Primary sources: Historic forest inventory maps (see Patek 2013)	Data used directly for 1954, 1976 and 2003; recalculation for years 1865 and 1988
Extensive grasslands, unused grasslands, unused/unproductive areas; settlement areas	Literature (SOM Table S3)	Literature on either C-stocks, or on NPP combined with turn-over times for different grassland types; secondary succession of woody plant species modelled
Croplands/intensive grasslands	Harvest statistics, literature (SOM Table S3)	Aboveground: croplands harvest extrapolated with “pre-harvest-loss” factors and mowing rates, belowground: literature data
NPP_act_		
Forests	Primary sources: Historic forest inventory maps (see Patek 2013, literature)	SC_act_ divided by stand age; expansion factors for litter
Shrublands	Primary sources: Historic forest inventory maps (see Patek 2013, literature)	SC_act_ divided by stand age in 2003; before constant 2003 SC_act_
Extensive grasslands; unused grasslands, unused/unproductive areas	Literature (SOM Table S3)	Literature on either C-stocks, or on NPP combined with turn-over times for different grassland types; secondary succession of woody plant species modelled
Croplands/intensive grasslands	Harvest statistics, literature (SOM Table S3)	Cropland harvest extrapolated with “pre-harvest loss factors”, multiple mowing events based on mowing factor
Settlement areas	1/3 of NPP_pot_	Modelled based on Haberl and others (2007)
HANPP_harv_		
Forests	Forest harvest statistics, literature on litter and forest grazing demand	Timber volumes converted to C-units, grazing and litter demand per livestock unit
Extensive grasslands	Livestock statistics, literature	Feed demand modelled through livestock numbers and feed demand values; literature on grassland biomass available for grazing
Croplands/intensive grasslands	Harvest statistics; literature	Cropland primary harvest extrapolated with harvest indices and recovery rates, literature
Settlement areas	1/2 of NPP_act_	Modelled based on Haberl and others (2007)

### Actual Carbon Stocks (SC_act_)

SC_act_ was mapped and quantified based on different approaches for the individual LULC-types. Forest SC_act_ maps were available from a previous study for the years 1834, 1947, 1976 and 2000 (Patek 2013). We assumed the 1947 map to represent the 1954 conditions, the 1976 map to represent the 1973 and the 2000 map to represent the 2003 conditions to be consistent with the LULC-maps. The forest SC_act_ maps had been compiled based on historic forest inventory maps (Von Guttenberg 1834; Mappenarchiv des Vermessungsamtes Innsbruck 1861; Landesforstinspektion für Tirol 1948, 1979, 2004) that contain information on timber volumes, applying the “stock change method” (IPCC 2003). Timber volumes had been converted to mass units using a standard factor of 0.4 tons dry matter per m^−3^ of timber volume for spruce forests (IPCC 2003). Solid timber volumes had been converted to total tree biomass, including roots, branches and leaves, using biomass expansion factors provided by Kramer and Krüger (1981). We calculated forest SC_act_ for the year 1865, assuming SC_act_ in 1834 to equal SC_act_ in 1865 and applied the mean SC_act_ of the years 1947 and 1834 to areas that were not forests in 1834. For the year 1988, no forest SC_act_ map was available. Hence, we used the arithmetic mean of the 1973 and 2003 SC_act_ maps for all forest pixels in the 1988 LULC map.

The 2003 forest SC_act_ map also contained areas that were classified as shrublands in the LULC map. We applied the average value of all such pixels for shrublands outside the forest SC_act_ map. For all remaining LULC-classes that have a much lower C-density (Saugier and others 2001; Erb 2004), we used data from the literature (SOM Table S3). The category “unused grasslands/shrublands” emerged in 1954, consisting mainly of abandoned grasslands at higher altitudes. We used the SC_act_ of extensive grasslands for this category in 1954, while we considered C-density increases afterwards: In case a pixel remained “unused grasslands/shrublands” until 2003, we assumed the average SC_act_ between extensive grasslands and shrublands for 1973, 1988 and 2003 to account for secondary succession of shrubs. If a pixel was unused grassland/shrublands in 1973, but forest land in 2003, the 1988 SC_act_ value was calculated as the mean between both years.

### Actual NPP (NPP_act_)

Forest NPP_act_ was calculated as the sum of woody NPP_act_ and NPP_act_ of needles and leaves. Woody NPP_act_ was calculated by dividing the total SC_act_ with forest age, which was available in the forest inventory maps (previous section). If age information was missing, we assigned the mean age of forests that had similar C-density. The NPP_act_ of needles and leaves was extrapolated from woody NPP, using age-specific biomass expansion factors for different Austrian forest types (SOM Table S4). The resulting values were divided by average needle age, which was assumed to be 7 years for montane and 10 years for sub-alpine spruce forests, as well as one year for deciduous forests and larch forests.

Because intensive grasslands are usually harvested more than one time a year (NPP_act_ > SC_act_), we multiplied NPP_act_ on intensive grasslands with a mowing-frequency factor (SOM Figure S2). NPP_act_ on annual croplands is strongly related to HANPP_harv_ (next section, refer to Erb and others 2009), and it was calculated by extrapolating HANPP_harv_ with temporally dynamic pre-harvest loss factors that account for NPP losses due to herbivory or weeding (Haberl and others 2007; Krausmann and others 2013). For modelling secondary succession on unused grasslands/shrublands, we applied the similar assumptions as for SC_act_ (previous section). NPP_act_ of infrastructure areas was calculated as 1/3 of NPP_pot_, assuming that two-thirds are sealed (Haberl and others 2007).

NPP_act_ on all remaining LULC-classes, mainly low-productive alpine classes, were assessed through literature research (SOM Table S3). To account for climate-related NPP increases on such areas, we applied the temporal dynamic derived by the MIAMI model (Lieth and Whittaker 1975), using historic temperature and precipitation data (Auer and others 2007). The MIAMI model is an empirical model that calculates NPP of a region as the minimum between NPP derived as a function of annual mean temperature and of annual precipitation. It is based on a global site-data network (Lieth and Whittaker 1975) and is still widely used in global change studies due to its simple calculation procedure and the overall robustness of results.

### Harvested NPP (HANPP_harv_)

Cropland HANPP_harv_ consists of primary crop harvest, for which statistical records were available and crop residues, which we modelled through crop-specific, temporally dynamic harvest factors (Gingrich and others 2015). Only province-level cropland data (cultivar mix, area, yield) were available (Central Office for Statistics 1869; Statistics Austria 2011), and thus we applied the Tyrolian spatial composition and yields of different crop types to the cropland areas in Neustift. Harvest of wood volumes was available from forest statistics (Landesforstinspektion für Tirol 1948, 1979, 2004). Because data for 1865 were missing, we used the average wood demand per household in a similar study region (Großarl in the Austrian Central Alps, 1835) by Krausmann (2008), assuming a household size of five persons. Roundwood volumes were converted into C flows assuming an average wood density of 0.405 t dry matter m^−3^ round wood and a C-content of 50% (Krausmann and others 2013). Harvested by-products (for example, bark, stumps, branches) were accounted for using a bark factor of 80% and a wood recovery rate (the ratio of removals to fellings) of 89% (Krausmann and others 2013). Until far into the twentieth century, harvest of forest litter for mulching and animal husbandry as well as forest grazing played an important role in most European forests (Eckmüller and others 1970; Stuber and Bürgi 2002; Emanuelsson 2009; Erb and others 2013b; McGrath and others 2015). We used the value of litter demand per livestock unit (Table S5) provided by Krausmann (2008) for Großarl, which considers an annual demand of roughly 380 kgC LU^−1^ a^−1^ through litter extraction. We assumed that litter extraction decreased in the second half of the twentieth century and that extraction in 1973 amounted to only half of the 1954 value. In the year 1975, the litter extraction had been forbidden per law and disappeared afterwards.

The amount of grazed biomass was assessed by combining different data sources. First, livestock feed demand was assessed by multiplying livestock numbers (Central Office for Statistics 1869; Statistics Austria 2011) with temporally dynamic and animal-specific feed demand values (Gingrich and others 2015). Next, available livestock fodder was quantified. HANPP_harv_ on grasslands was calculated based on their annual productivity (Egger and others 2005; Tasser and others 2012), considering the length of the growing season, derived as a function of elevation and climate conditions (Harlfinger and Knees 1999), a topographical correction factor that depends on slope and aspect and the amount of summer mean precipitation. For alpine meadows, we considered a grazing level of 25% of the HANPP_harv_ per area of extensive grasslands.

We used the value of grazed biomass per forest area provided in Krausmann (2008) for Großarl to derive HANPP_harv_ through forest grazing in 1865 and in 1954. Forest grazing had been forbidden by 1975, which is why we assumed the 1973 grazing pressure to be half of the 1954 level and did not consider forest grazing afterwards. Following previous studies, we also considered 50% of NPP_act_ on infrastructure areas to be harvested through gardening (Haberl and others 2007). Eventually, we assumed that the difference between overall feed demand and the sum of all mentioned feed sources was extracted from croplands, or that it was imported.

### Potential C-Stocks (SC_pot_) and NPP (NPP_pot_)

We used an existing map of potential vegetation, which depicts a hypothetical land cover after maximum land abandonment (Tasser and others 2017). Tasser and others (2017) had calculated a potential treeline using a GIS approach and statistical applications. Based on the assumption that the potential tree line is more elevated than the actual treeline, they selected the highest forest tree lines in the available LULC-maps and derived functions to describe the correlation among important site characteristics (altitude, aspect, slope) by means of polynomial regressions. We used this map and reclassified the small remaining plots of infrastructure and agriculture into low-land alluvial forests in the valley, or into montane (<1600 m a.s.l.) or sub-alpine forests at higher altitudes.

We modelled forest SC_pot_ and NPP_pot_ based on the strong negative linear relationship between the upper 20 percentiles of forest pixels in 2003 and rising altitude (SOM Figure S1) using a digital elevation model provided by the Tyrolean Information System tiris (Land Tirol©). For low-land alluvial forests, we considered the average of the upper 20 percentiles of SC_act_ of all forest grid cells occurring up to 1000 m a.s.l. In the remaining LULC-classes, we considered potential SC and NPP to equal the 2003 conditions. We applied the temporal trend of the MIAMI model (refer to chapter 2.2) to account for NPP_pot_ increases owing to climatic changes. Because this effect on SC_pot_ is unclear and can potentially result either in SC-increases or in a shortening of the C residence time in ecosystems (Körner and others 2005; Malhi 2012), SC_pot_ was held constant over time.

#### HANPP

HANPP was calculated on basis of the described NPP categories, following the definition by Haberl and others (2007) and Erb and others (2009):

1 $$ \text{HANPP} = \text{HANPP}_{{\text{harv}}} + (\text{NPP}_{{\text{pot}}} - \text{NPP}_{{\text{act}}} ) $$

### Auxiliary Data

As socio-economic background data, we gathered data on population numbers (Statistics Austria 2015) and people employed in agriculture. For 1865 and 1954, we used the Tyrolian average due to lack of data (Möller 1974). Because data were missing, we used the national average for nitrogen (N) consumption and tractor numbers per area of cropland as indicators for input-intensification (Dachs and others 1997; Statistics Austria 2015). As an input-intensity metric that was particularly important in pre-industrialized times, we also calculated manure produced by livestock, following the approach in Haberl and others (2007), which considers a fraction of 35% of the C-intake of cows and 25% of all other animal species to be excreted. Of this amount, 1/3 is subtracted, assuming that a certain share of dung is not available on the fields (Haberl and others 2007).

## Results

**Figure 1:**
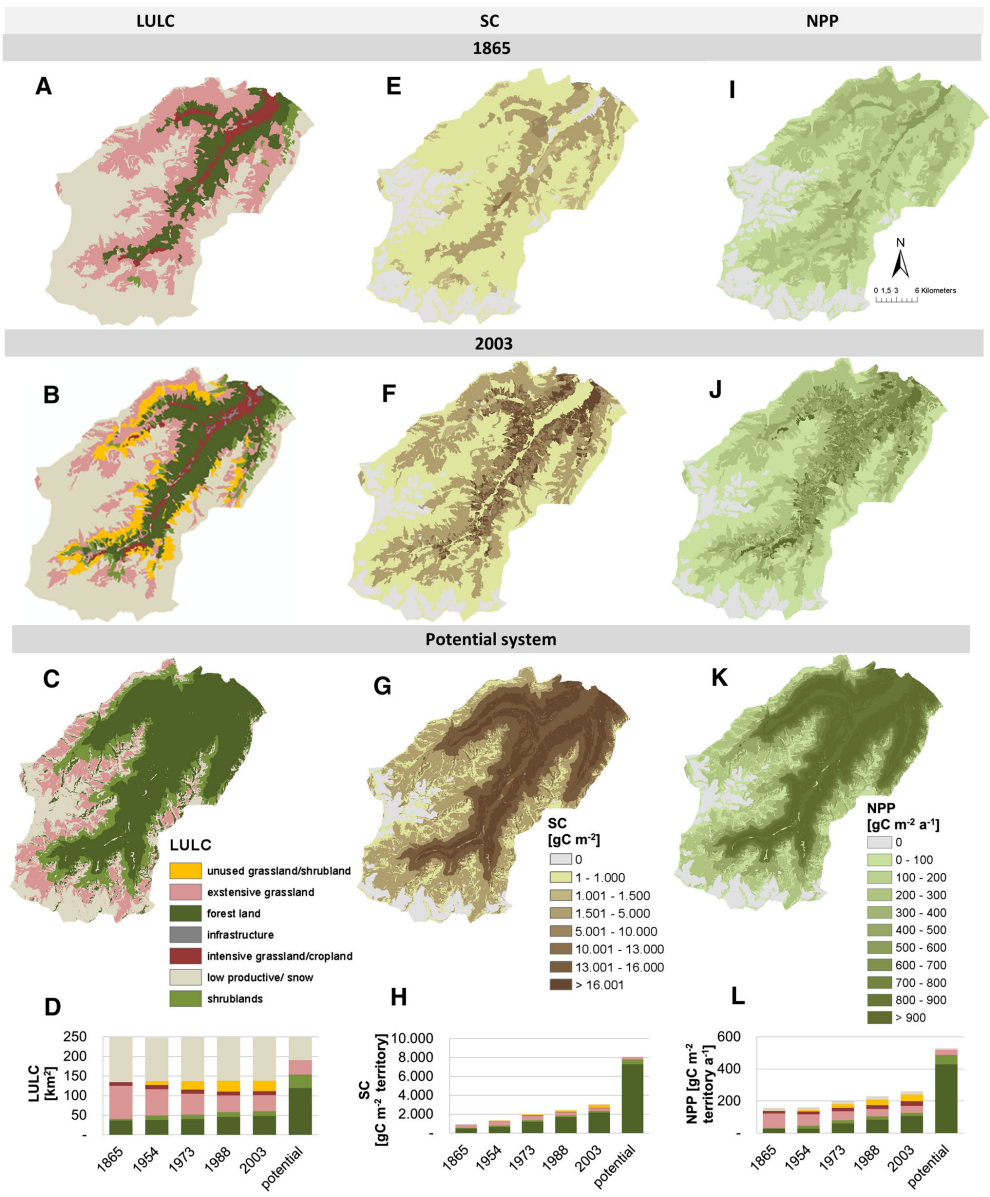
Patterns of LULC (**A**–**C**), SC (**E**–**G**) and NPP (**I**–**K**) in the Stubai valley. *Maps* in the *first row* show patterns for the year 1865, the *second row* for 2003 and the *bottom maps* show the potential land system. (**D**) Trends of LULC-changes for all time cuts (1865, 1954, 1973, 1988, 2003) broken down to the main LULC-classes, (**H**) SC and (**I**) NPP values for all time cuts normalized by the total study area. *Colour codes* of the LULC legend (**C**) matches the *colour codes* of** D**,** H** and** L** (Color figure online).

Spatial patterns of LULC, SC_act_ and NPP_act_ differed markedly between the years 1865, 2003 and the potential vegetation (Figure 1), being strongly dominated by forest dynamics. SC_act_ more than tripled from 939 to 3050 gC m^−2^ between 1865 and 2003 (Figure 1H), and NPP_act_ grew by 65%, from 158 to 262 gC m^−2^ a^−1^, experiencing the most rapid increases after 1954 (Figure 1D, H). Between 1865 and 2003 extensive grasslands declined by 51%, whereas forests increased by 30% (Figure 1D). Unused grasslands/shrublands doubled between 1954 and 1973 and since then remained at a rather constant absolute level, covering ca. 10% of the territory. The slight decrease after 1988 is related to the establishment of secondary forests (refer to the SOM Table S6). Ample LULC-changes after 1954 resulted in an apparent peak of area changes between 1973 and 1988, both in terms of net- as well as gross changes (Figure 1D, SOM Figure S3). This was chiefly dominated by expansion of unused grasslands/shrublands at the cost of extensive grasslands and by forest regrowth on unused grasslands/shrublands (SOM Figure S3, Table S6).

NPP patterns closely resemble SC patterns, with the exception of intensive grassland/croplands, which are characterized by NPP_act_ in all time steps (Figure 1I, J, L). Despite only covering 4% of the territory, this LULC-type contributed 11% to NPP_act_ in 2003. SC_pot_ and NPP_pot_ levels were still 62 and 51% larger than the SC_act_ and NPP_act_ in 2003 (Figure 1H–L).

All LULC-types experienced increases of both, SC_act_ and NPP_act_, except intensive grassland/croplands, where SC_act_ remained rather stable, as well as extensive grasslands, where NPP_act_ remained stable (Table 2). SC_act_ and NPP_act_ increases were most prominent on forest land, experiencing a growth from 3 to 11 kgC m^−2^ (increase by 238%) and from 187 to 551 gC m^−2^ a^−1^ (194% increase), respectively (Table 2). NPP_act_ increases were also pronounced on intensive grasslands/croplands (increase by 76%), the LULC-type with the highest average NPP_act_ level (730 gC m^−2^ a^−1^) in 2003. Unused grasslands/shrublands reveal moderate levels of NPP and higher SC_act_ than extensive grasslands in 2003.

**Table 2:** SC_act_, and NPP_act_ in 1865 and 2003 Broken Down to the Main LULC-Types

		Forest land	Shrublands	Extensive grasslands	Intensive grass-/croplands	Infrastructure	Unused grasslands/shrublands	Low productive/alpine	Total
SC_act_ (gC m^−2^)	1865	3316	2939	1016	828	202	0	51	939
	2003	11 217	3489	1808	803	382	3336	86	3050
NPP_act_ (gC m^−2^ a^−1^)	1865	187	172	272	414	264	0	45	158
	2003	551	413	268	730	290	389	47	262
SC-gap (1000 tC) (% of total gap)	1865	513 (29)	53 (3)	751 (42)	124 (7)	2 (0)	0	334 (19)	1777 (100)
	2003	284 (23)	128 (10)	210 (17)	125 (10)	17 (1)	169 (14)	318 (25)	1251 (100)

The SC-gap indicates the distance between SC_act_ in 1865 and in 2003 to the potential system, using the 1865 and 2003 LULC-masks.

SC_pot_ and NPP_pot_ levels presented in Table 1 refer to the average potential levels of the 2003 LULC map. Forest land shows by far the highest levels of 17 kgC m^−2^ SC_pot_ and more than 1000 gC m^−2^ a^−1^ NPP_pot_, followed by intensive grasslands/croplands, shrublands and infrastructure areas. Unused grasslands/shrublands and extensive grasslands reveal moderate levels of SC_pot_ and NPP_pot_, whereas low productive/alpine regions show the lowest level of roughly 3 kgC m^−2^ and 227 gC m^−2^ a^−1^, respectively.

The absolute difference between SC_act_ and SC_pot_ (SC-gap, Table 2) declined from 1.8 Mio tC in 1865 to roughly 1.3 Mio tC in 2003, which represents a decline from 88 to 62% of SC_pot_. This difference was highest on extensive grasslands in 1865, which contributed 42% (751 ktC) to the found SC-gap. However, its contribution decreased notably to only 17% in 2003, when low productive/alpine regions contributed the highest share of 25%, although their absolute C-gap, that is, around 318 ktC, remained relatively stable (Table 2). In 2003, unused grasslands/shrublands contributed 14% to the SC-gap in 2003, showing the long legacy effects of stock depletions. In contrast, infrastructure areas, intensive grasslands/croplands and shrublands both contributed 10% to the total SC-gap, where the SC-gap on shrublands almost tripled from 1865 to 2003, while it remained rather stable on intensive grasslands/croplands. Forest areas, despite increasing so drastically in terms of their C-density, still contributed 23% to the SC-gap in 2003 (Table 2).

**Figure 2:**
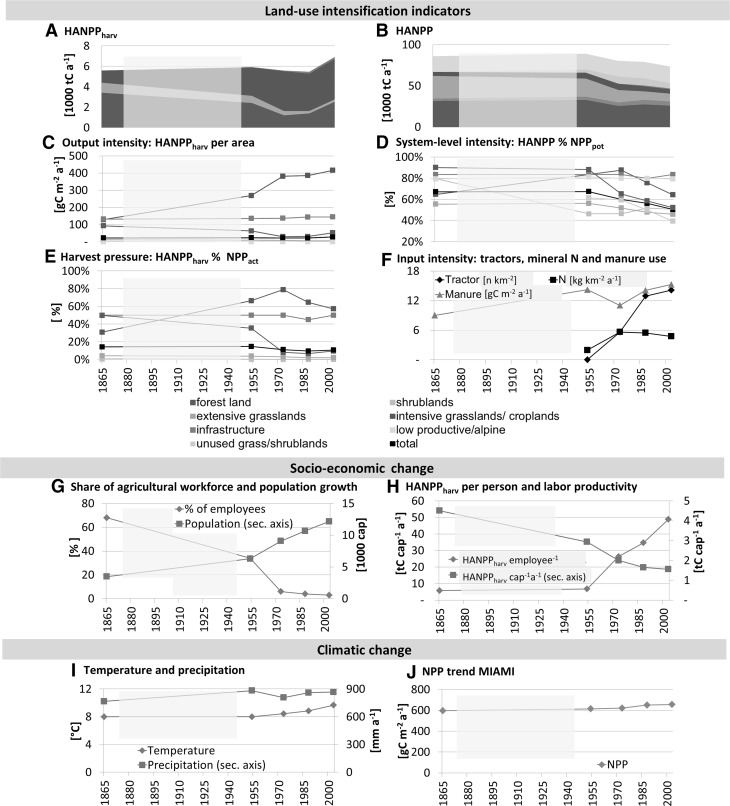
Land-use intensity trends, socio-economic changes and climatic changes from 1865 to 2003: **A** harvested biomass (HANPP_harv_) and **B** HANPP in 1000 tC a^−1^; **C** HANPP_harv_ per area, **D** HANPP as percentage of NPP_pot_; **E** share of NPP_act_ harvested per year (HANPP_harv_ % NPP_act_); **F** tractor numbers and nitrogen (N) inputs per area of intensive grasslands/croplands (only Austrian levels available) and manure per area of agricultural land (intensive grassland/cropland plus extensive grasslands); **G** people employed in agriculture as share of the working population and population numbers (secondary axis); **H** HANPP_harv_ per agricultural worker and per person (secondary axis); **I** temperature and precipitation (secondary axis) data for Innsbruck city, **J** NPP trend based on the MIAMI model calculated with data from (**I**). The semi-transparent area between the *first* and *second time-cut* indicates lacking data, trends between 1865 and 1954 were linearly interpolated.

Figure 2 illustrates changes in land-use intensity indicators, socio-economic changes and climatic changes that were underlying drivers of the detected land system patterns. Total biomass harvest (HANPP_harv_) remained remarkably stable between 5.5 and 5.9 ktC a^−1^ over the time period, experiencing a growth only by 2003 due to rising forestry harvest (Figure 2A). Major shifts occurred in the composition of LULC-types contributing to HANPP_harv_ (Figure 2A). The contribution of forestry harvest declined from 61% of total harvest in 1865 to only 37% in 2003. In contrast, the contribution of intensive grasslands/croplands increased substantially from 21% in 1865 to 57% in 2003. Here output intensity, measured as HANPP_harv_ per area, more than tripled between 1865 and 2003 (Figure 2C), resulting in an increase in the share of HANPP_harv_ per NPP_act_ from 31% to 57% (Figure 2E). In other words, harvest efficiency increased. Forest areas experienced the opposite trend, revealing a decline of output intensity (Figure 2C) and a drastic reduction in HANPP_harv_ from 50 to 10% of NPP_act_ (Figure 2E). These overall increases in HANPP_harv_ did not result in an increase in HANPP, but were compensated for by increases in NPP_act_, allowing for declining absolute as well as relative HANPP levels (Figure 2B, D). Although in 1865 HANPP amounted to 67% of NPP_pot_, it was only 51% in 2003 (Figure 2D, black line). This was mainly caused by the dynamics of forests and extensive grasslands.

N-inputs almost tripled between 1954 and 1973 and slightly declined afterwards, whereas tractor numbers per area of intensive grasslands/croplands increased from 0.02 to over 14 tractors per km^2^ (Figure 2F), indicating a trend towards mechanization and industrialization of agriculture. Manure production increased steadily owing to rising livestock numbers and changing livestock composition (Figure 2F).

Socio-economic changes acted as underlying drivers of the described changes in land-use intensity and SC. The main changes were (a) structural economic change, that is, a shift towards industry and manufacturing as primary income sources (Figure 2G) and (b) a decreasing significance of biomass in the local economy. Although population numbers grew 3.5-fold between 1865 and 2003 (Figure 2G), the agricultural workforce declined from 68 to 3% of the working population (Figure 2G). At the same time, agricultural labour productivity (HANPP_harv_ per agricultural worker, Figure 2H) increased drastically. One person produced ca. 6 tC of HANPP_harv_ per year in 1865 and 49 tC in 2003. In contrast, biomass extraction per person decreased drastically, which is indicated by HANPP_harv_ that declined from 4.5 to 1.6 tC a^−1^ (Figure 2H). Climatic changes were also pronounced in the study region. The rise of average annual precipitation and temperature over the past 140 years (Figure 2I) led to a 10% increase in the NPP_act_ level calculated through the MIAMI model (Figure 2J).

**Figure 3:**
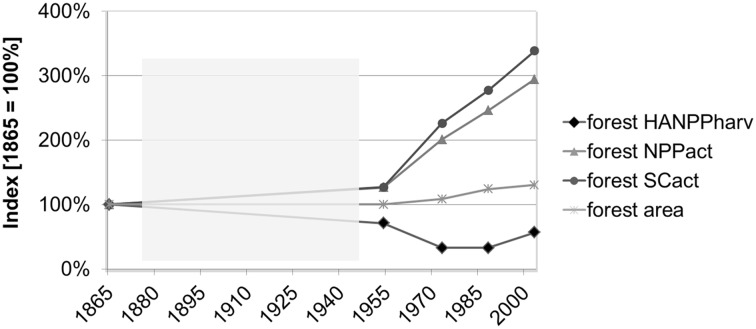
Forest dynamics (Index 1865 = 100%): Forest HANPP_harv_, forest NPP_act_, forest SC_act_ and forest area trends from 1865 to 2003. The *semi-transparent areas* indicated missing data and a linear interpolation of trends.

Forest SC_act_ and NPP_act_ experienced a drastic take-off after 1954, rising by more than 300% until the end of the study period. In contrast, forest HANPP_harv_ decreased from 1954 to 1973, but increased again to about the 1954 level in 2003 (Figure 3). In contrast, forest expansion was moderate and slightly levelled off between 1988 and 2003.

## Discussion

### Studying 140 Years of Land System- and C-Dynamics

In this study, we investigate the role of land-use intensification for SC_act_ changes in the municipality of Neustift in the Stubai valley, Austrian central Alps, from 1865 to 2003. The extremely rich data basis allows us to scrutinize the role of different LULC-types for C-accumulation in the past 140 year, when SC_act_ tripled from 3.3 to 11.2 kgC m^−2^. The HANPP framework, providing a robust reference measure (NPP_pot_) and allowing for integrative analysis across different land-use types, proved useful for disentangling the role of area versus land-use intensity changes on this massive increase in SC, particularly on forest land. The HANPP framework was suitable for revealing the systemic interlinkages between different land-use types, for example, effects of intensification of one LULC-type (intensive grasslands/cropland) allowed for C-accumulation in another one (forests). In the following sections, we discuss these findings separately for the two distinct phases of land system change we observed over the past 140 years. We continue with a discussion on the SC-gap to the potential system and finish with a short reflection of data uncertainties und limitations of our analysis.

### High Land-Use Impact and Forest Depletion: 1865–1954

Socio-economic changes in Neustift over the past 140 years were fundamental and played out in terms of striking shifts of LULC patterns and SC_act_ levels (Figure 1). Compared to 2003, the 1865 SC_act_ level of 3.3 kgC m^−2^ was low, indicating a high land-use impact. Similar SC_act_ levels are typically reached in global ecosystems such as scrublands and tree-bearing natural grasslands (Saugier and others 2001), whereas the 2003 level of 11.2 kgC m^−2^ compares well to typical values for temperate forests.

HANPP levels in 1865 indicate a high land-use pressure on all LULC-types (Figure 2B, D), where particularly the intensive use of forests resulted in low NPP_act_ and SC_act_ (Figure 2B; Table 2). The forest and livestock systems were closely interlinked through NPP flows until far into the twentieth century, which is manifested in a high share of forest HANPP_harv_, between 20 and 30% entering the livestock system as either litter or grazed biomass until 1954. This led to a substantial harvest pressure of 50% of NPP_act_ in forests by 1865 (Figure 2E), which is clearly beyond the sustainability thresholds and supports the notion of severe forest degradation related to grazing and litter extraction found in other parts of Europe (Stuber and Bürgi 2002).

By 1954, forest areas had already started to increase slightly over the 1865 level, and small shares of extensive alpine grasslands had been abandoned from use and structural economic change had already withdrawn more than 60% of the working population from agriculture to industry, or services (Figure 2E). However, the 1954 land-use intensity and SC_act_ patterns still resemble many characteristics of the nineteenth century, that is, high HANPP levels and low forest SC_act_, which is partly related to the high fuel-wood demand around World War II (Landesforstinspektion für Tirol 1948; Gold and others 2003, 2006). Although output-intensification in 1954 (Figure 2E, F), demonstrated by rising HANPP_harv_ per area (Figure 2C), had already started to increase, input-intensification was still low compared to the consecutive period (Figure 2F) and labour productivity was ca. at the 1865 level (Figure 2F).

### Polarization and C-Accumulation: Post-1954 Land System Trends

It was only after 1954 that the land system in Neustift shifted towards the characteristics of a fully industrialized system, late in comparison with other European countries (Jepsen and others 2015). Many of the observed trends after 1954 are in line with conceptual notions on the drivers of the so-called forest transition (Mather and others 1999; Erb and others 2008; Gingrich and others 2015), basically founded in a land-use regime shift (Krausmann and others 2008; Jepsen and others 2015; Gingrich and others 2016). Although the pre-industrial system was in principle relying on solar energy, mainly in the form of biomass, the abundance of an area-independent energy source, fossil energy, alleviated land-use pressures. In this period, polarization into intensive and extensive LULC-types emerged as the new paradigm in Neustift, as in other European regions (Jepsen and others 2015). Compared to the 1865–1954 period, LULC-changes were much more dynamic, being chiefly dominated by rapid abandonment of extensive grasslands at higher altitudes and afforestation on unused areas, particularly from the mid-1980s onwards (Figure 1A–D; SOM Figure S3). These trends are reflected by drastically decreasing HANPP levels that allowed for accumulating SC_act_ (Figures 2E, 3).

Surging NPP_act_ after 1954 was central for this witnessed decline in HANPP, despite HANPP_harv_ increased (Figure 2A, B). NPP_act_ increases emerged as the combined effects of changing climatic and socio-economic drivers. Rising temperature and precipitation levels led to a climate-induced NPP_act_ increase of 10% between 1865 and 2003 (Figure 2I, J), which, however, only partly explains the 65% increase we observed. This hints at socio-economic drivers that dominated the observed land-use processes and NPP_act_ trends. Rising input-intensification through tractors and mineral fertilizers (Figure 2H) allowed NPP_act_ and HANPP_harv_ to triple on intensive grasslands and croplands (Figure 2F). Related to that the forest and livestock systems were gradually disentangled, because intensive grasslands/croplands, though small in spatial extent, supported a rising share of animal fodder (Figures 2F, 3). This together with the availability of concentrated feed stuff and a legal prohibition on litter extraction (Landesforstinspektion für Tirol 1948, 1979, 2004) contributed to reduced harvest pressure on forests (Figure 2E). The amount of forest grazing and litter extraction had shrunk to zero by 1988.

Interestingly, the increase in forest HANPP_harv_ in 2003 is not reflected in the forest SC_act_ trend (Figure 3). This indicates that in addition to the total amount of forest HANPP_harv_, the type of extracted biomass plays a decisive role for C-accumulation. Forest grazing and litter extraction resulted in a drastic reduction of the reproductive parts of forests (leaves, small branches), which hindered high NPP_act_ and SC_act_ levels until 1954. The focus was more on harvesting the structural components afterwards (timber), which is why SC_act_ and NPP_act_ could increase, despite absolute forest harvest remained rather constant (Figures 2E, 3). Hence, more importantly than changes in forest areas, the amount and in particular the type of forest harvest strongly influenced forest harvest pressure and SC_act_ levels in the study period.

The altering role of forests as hotspots of decreasing HANPP and C-density increases resembles the fundamental changes in the Austrian energy sector after WWII (Krausmann and others 2009) that allowed for population increases in Neustift, without increasing wood harvest (Figure 2G, A). In line with the general trend found in European forests, forests in Neustift became older and denser (Nabuurs and others 2003; Vilén and others 2012) and ecosystem services provided by forests shifted from primarily energy provision, to protection (Landesforstinspektion für Tirol 1979) and recreation (Pröbstl and others 2010).

Structural changes of the economy went on rapidly after WWII, and many farmers shifted to off-farm employment in industry or service- often tourism (Tappeiner and others 2008; Schermer and others 2016), whereas the share of people employed in agriculture declined markedly (Figure 2G). Labour efficiency increased steadily, where HANPP_harv_ cap^−1^ a^−1^ tripled between 1954 and 2003 (Figure 2H). Because cropland and livestock production was more and more concentrated in the low-land intensive LULC-classes, extensively used areas at higher altitudes were increasingly abandoned from use (Figure 1, SOM Figure S3). In 1973, 10% of the territory, mostly former extensive grasslands, consisted of unused grasslands/shrublands, which increased their C-densities from around 800 gC m^−2^ in 1954 to 3300 gC m^−2^ in 2003 as a result of woody encroachment.

### The C-State: Comparisons to the Potential System

Despite the marked SC_act_ and NPP_act_, increases witnessed over the past 140 years in Neustift, NPP and SC levels in 2003 were still well below the potential system, ranging up to only 38 and 49% of the potential level, respectively (Figure 1H, L). This indicates a substantial land-use impact in 2003, despite the observed trends of recovery from past forest depletion, an overall reduction in harvest intensity and declining HANPP. Conversion of potential land cover, mostly forests (Figure 1D), to grasslands, croplands, or settlement areas, accounted for the lion’s share of 77% of the SC-gap in 2003 (Table 2) and concerned roughly 57% of the study area. The remaining SC-gap is attributed to forest areas in 2003, which had not experienced any change in potential land cover (Table 2), highlighting the central role of forest management on SC-reductions. Only 23% of the study area did not experience either management, or land cover changes and as such did not contribute to the C-gap.

Our results support the significance of integrating effects of past land-use legacies and effects of time-lags between the period land-use changes occurred and when a new SC-climax is established (Bürgi and others 2016). The relative peak of changes in areas found in our study (Figure 1D, SOM Figure S3) and in Tappeiner and others (2008) between 1973 and 1988, as well as the concomitant peak of output intensity by 1988 (Figure 2E), is not reflected in the trends of SC_act_ and NPP_act_ that continued to increase unhalted until 2003. The vegetation in Neustift is still net-accumulating C as a consequence of land-use processes that have happened years, or decades ago. Hence, the SC-gap to the potential system will likely further decrease in the coming years. Assuming that on LULC-types that were not under use by 2003 (shrublands, unused grasslands/shrublands, low productive/alpine areas) SC_pot_ will be re-established, the current SC-gap could be halved in the coming decades.

### Data Uncertainty and Limitations of Analysis

We relied on several assumptions on how to use empirical data for the time-series reconstruction in this study, which might have introduced uncertainty. As we paid particular attention to the contingency of methods, and the HANPP framework has been shown to yield reliable and robust time-series results despite large uncertainties (Krausmann and others 2013), our detected trends are more robust than the absolute levels of certain parameters. One example is the calculation of grazing demand, which was based on Austrian average animal-specific feed demand values, which might have been too high for Neustift and could have led to an over-estimation of grazed biomass. In addition, we could not depict the effect of specific land uses due to a lack of data. For instance, it was not possible to assess the impact of transhumance grazing by livestock that were not living in the study area permanently and thus were not accounted for in the statistics. Note that effects of these two data problems might partly be outbalanced in our overall results. Also, the NPP_pot_ and NPP_act_ trend estimate derived by the MIAMI model, used to assess, for example, dynamics on extensive grassland LULC-types can serve as a proxy only, because the MIAMI model only considers precipitation and temperature, but not changes in atmospheric CO_2_ over the study period (Sitch and others 2003). This could have led to overestimations of historic NPP levels. However, we believe that these effects have a minor influence on our results, because NPP changes in forests and intensive LULC-classes are not model-derived and thus indirectly include the effect of elevated CO_2_.

In this study, we used NPP flows as metrics for land-use intensity and as such did not quantify other important aspects of intensification, such as changes in the nitrogen or phosphorus cycles that can tremendously influence C-dynamics and the state of ecosystems (Bouwman and others 2011). In the background of the central role of terrestrial ecosystems as sinks or sources of atmospheric carbon (Le Quéré and others 2015), understanding the role of such biogeochemical processes will be key for advancing our knowledge on the determinants of terrestrial SC.

The main insights of our study were particularly derived by a quantification of forest management effects on SC_act_. Thus, our results deviate from the results by Tappeiner and others (2008), who used constant SC_act_ per unit area for forests and quantified a SC_act_ increase by only about 21% for the same study area, in contrast to the tripling we found. Our results are also in line with the findings on massive SC_act_ per unit area increases in central European forests in the last decades and centuries (Gold and others 2006). The assessment of soil organic carbon (SOC) changes were beyond the scope of our study. The available database did not allow us to depict effects of land-use changes on this crucial parameter. The literature is inconclusive on the effect of management on SOC changes (Lettens and others 2004; Schulte and others 2006; Wiesmeier and others 2013), and field data in the study region have shown that SOC is highly site-specific (Mantl 2013). This makes an upscaling to the regional level intricate, particularly if the often very long legacy effects of past land use are taken into account (Gimmi and others 2013; Bürgi and others 2016). Modelling studies show that SOC effects have a long time-delay, but might be of smaller importance at the landscape level than biomass changes (Pongratz and others 2009).

It is difficult to judge to what extent our results are relevant for other mountainous regions in the world that saw similar shifts in the energy system along with industrialization. It might be suspected that the substitution of land-based energy carriers with fossil energy and a decreasing role of biomass allowed for recovery from past forest depletion also in those regions, as the wide-spread occurrence of a “forest transition” suggests (Kauppi and others 2006; Erb and others 2008). For non-mountainous areas, SC_act_ increases could be more moderate than we find for Neustift. For instance, the Austrian average, revealed a much lower SC_act_ increase in 20% from 1830 to 2000 (Gingrich and others 2007), which illustrates the peculiar dynamics of mountainous regions.

## Conclusions and Outlook

Our study on land-use intensification and its effects on SC_act_ trajectories in the municipality of Neustift from 1865 to 2003 yielded several important insights. The HANPP framework, allowed us to assess land-use intensity as well as area changes across different LULC-types robustly in a historic time-series. Applying it to Neustift, we were able to show the highly-interlinked land-use processes that led to massive increases of C-stocks in the study region, despite biomass harvest increased. The HANPP framework proves useful for analysing underlying land-use processes and their individual roles that determine the dynamic of the C-state of ecosystems. For instance, we were able to identify the disintegration between the forestry and the livestock sector, both strongly linked through NPP flows until far into the twentieth century, as one of the most important determinants for forest SC_act_ increases. This effect is even stronger than the effect of expanding forest areas in the region. Our study provides evidence that land-use intensity and forest management are central components of the C-cycle and intrinsically interlinked with the capacity of ecosystems to act as vegetation C-sinks (Jandl and others 2007; Campioli and others 2015; Erb and others 2016a). This calls for a close consideration of such effects when planning for low-C societies.

## Electronic supplementary material

Below is the link to the electronic supplementary material.

Supplementary material 1 (RTF 13203 kb)
