# Supplementary material for: Influence of Land-Use Intensification on Vegetation C-Stocks in an Alpine Valley from 1865 to 2003
Source: Ecosystems. 2017 Mar 10;20(8):1391–406. doi: 10.1007/s10021-017-0120-5 (PMC6956954; doi:10.1007/s10021-017-0120-5)
Supplement: Supplementary file 1 — Supplementary material 1 (RTF 13203 kb) [file 10021_2017_120_MOESM1_ESM.rtf]

Supplementary Online Material 
Article: Influence of land use intensification on vegetation C-stocks in an Alpine valley from 1865 to 2003. 

Authors: Maria Niedertscheider, Erich Tasser, Monika Patek, Johannes Rüdisser, Ulrike Tappeiner, Karl-Heinz Erb
*corresponding author: maria.niedertscheider@aau.at, tel.: ++43 (0) 1 522 4000 342

Published in Ecosystems 


1.	Supplementary tables and text 

1.1 Aggregation of land use/cover (LULC) types 
All calculations are based on the original 24 land use/cover (LULC) classes provided in Tappeiner et al. (2004). For the Results (chapter 3) and Discussion (chapter 4) sections in the main text we used the following aggregation levels to derive seven aggregated LULC-classes (Table S1): 
Table S1: Aggregation of the 24 original LULC categories (on which all calculations are based on), to the seven land use classes that are shown for reasons of simplicity and clarity as results. 
Aggregated land use category 	Land use/cover category 	
Forest land 	Sub-alpine coniferous; Montane coniferous; Alluvial forests; Mixed forests	
Shrublands 	Larch shrubs; green alder shrubs	
Extensive grasslands 	Extensive grasslands; Larch meadows; Alpine meadows and dwarf shrub communities; Medium-intensity grasslands	
Intensive grasslands/ croplands	Intensive grasslands; Annual and permanent croplands 	
Infrastructure	Settlement areas; Parking lots	
Unused grass/shrublands 	Abandoned areas	
Low productive/alpine 	Snow/ice; Rock communities higher altitudes; Rock communities lower altitudes; Scree communities higher altitudes; Scree communities lower altitudes; Water bodies; Marshland; Riparian areas  	

1.2 Calculation of SCact and NPPact 
Biomass expansion factors were  used (Table S2) for different forest age classes in order to calculate total tree biomass out of timber stocks (refer to main text chapter 2.1). 
Table S2: Biomass expansion factors for different forest age classes after Kramer and Krüger (1981), which are used as multipliers for timber stocks in order to calculate total tree biomass that included not only the merchantable parts, but also roots, branches, leaves. 
age [a]	biomass expansion factors	
0-20	-	
21-40	2.10	
41-60	1.63	
61-80	1.50	
81-100	1.49	
101-120	1.46	
121-140	1.44	
141-160	1.45	

C-stock (SCact) values were collected for all original LULC-types and NPPact. All LULC-classes except forests, shrublands and croplands (refer to the main text chapter 2.1) were taken from Tappeiner et al. (2008) who derived SCact values for the Stubai-valley based on intensive literature recherché. Table S3 shows the original LULC classification and the respective LULC classification used in this study. In case LULC-classes are assigned several times to respective classes in Tappeiner et al. (2008), this indicates that the average value of these classes was taken. NPPact was calculated based on SCact values divided by average residence times for belowground (b) and aboveground (a) compartments. For alpine meadows NPPb data was available (Bahn et al. 2006) and we calculated the residence times by dividing bSCact with NPPb data and, and due to a lack of data, considered a-residence time to equal b-residence time for the calculation of NPPact. Since we lacked NPP data for rock communities and scree slopes and because SCact was already very low in these areas, we considered NPP to equal SCact here. For (1) extensive pastures and (2) intensive grasslands we used the b-residence time of grasslands under (1) low grassland management at around 2100 m.a.s.l and (2) high to medium grassland management at around 1500 m.a.s.l. provided by Leifeld et al. (2015). For extensive grasslands we considered an a-residence time of two years, which is about the average of intensive grasslands and alpine meadows. 
For NPPact of larch meadows we compared the average SCact value of larch meadows given by Patek (2013) for the year 2003 with SCact values for different age classes of larch meadows and derived a mean age of roughly 45 years (Rubatscher et al. 2006). We calculated the NPPact by dividing the SCact value by age and added NPP of understorey vegetation provided by Rubatscher et al. (2006). 
Table S3: SCact values of the LULC-classes based on Tappeiner et al. 2008, and residence-times used to derive NPPact. 
Tappeiner et al. 2008 	´This study 	SCa
[gC m-2]	SCb [gC m-2]	SC [gC m-2]	Residence time of SCa [a] 	Residence time of SCb [a]	NPPa [gC m-2 a-1] 	NPPb [gC m-2 a-1]	 NPP [gC m-2 a-1]	
Rural settlement1	Settlement areas 	63.9	186.9	250.8						
Other public facilities and infrastruct-ures1	Settlement areas	5.5	16.1	21.6						
Extensively used pastures, acid soils	Extensive grasslands 	214.8	627.8	842.6	22	4.23	107.4	150.33 	 257.7	
Extensively used pastures, calcareous soils	Extensive grasslands	121.1	803.3	924.4	22	4.23	 60.53	192.33	 252.8	
Traditionally used grassland	Grasslands, medium intensity 	180.0	937.8	1117.8	1.5	4.23	119.9	224.53	 344.5 	
Intensively used grassland
	Intensive grasslands	143.1	580.5	723.6	1	2.12	143.1	278.6	421.7	
Larch and orchard meadows 1	Larch meadows	6718.5	1775.5	8494.0						
Acid rocks, alpine-nival zone1	Rock communities higher altitudes, Rock communities lower altitudes	9.7	29.0	38.6						
Acid scree slopes, alpine-nival zone1	Scree communities higher altitudes, Scree communities lower altitudes	26.8	18.9	45.7						
Calcareous rocks, alpine-nival zone1	Rock communities higher altitudes, Rock communities lower altitudes	9.9	24.6	34.5						
Calcareous scree slopes, alpine-nival zone1	Scree communities higher altitudes, Scree communities lower altitudes	15.3	16.0	31.3						
Acid rocks, alpine-nival zone1	Rock communities higher altitudes, Rock communities lower altitudes	14.7	29.0	43.7						
Calcareous rocks, alpine-nival zone1	Rock communities higher altitudes, Rock communities lower altitudes	90.7	84.8	175.5						
Acid scree slopes, alpine-nival zone1	Scree communities higher altitudes, Scree communities lower altitudes	23.3	24.6	47.9						
Calcareous scree slopes, alpine-nival zone1	Scree communities higher altitudes, Scree communities lower altitudes	120.2	68.4	188.6						
Alpine meadows, acid soil	Alpine meadows and dwarf shrubs	321.5	791.4	1112.8	4.2	4.2	77.0	189.43	 266.4 	
Alpine meadows, calcareous soil	Alpine meadows and dwarf shrubs 	271.0	606.9	877.9	3.2	3.2	84.6	189.43	 274.0 	
Dwarf shrub communitie, calcareous soil	Alpine meadows and dwarf shrubs	572.0	887.3	1459.3	7.1	7.12	79.3
	170.3 
	249.6
	
Dwarf shrub communitie, acid soil	Alpine meadows and dwarf shrubs	566.4	1216.2	1782.6	7.1	7.12	 56.6 	170.3	 226.9	
Wetlands, moors	Marsh land	126.2	4014.7	4140.9	1	0	126.2	126.2 	 252.3	
1 Other method than through residence-time was used to derive NPP values. See text to the table, as well as main text. 
2 Residence time taken from Leifeld et al. (2015)
3 NPPb data taken from Bahn et al. (2006)
The following factors (Table S4) were used for the calculation of NPP of needles in different forests (refer to the main text chapter 2.3) 
Table S4: Share of needles to total tree biomass [%] used to derive NPP of needles and leaves from SCact (refer to main text). Source: Kramer and Krüger (1981). 
Forest Age	Spruce	Pine	Beech	
[a]	[%]	
10	56	31	35	
30	19	13	17	
50	9	10	3	
65	7	8	4	
80	7	9	2	
110	7	6	3	

Litter demand (refer to the main text chapter 2.3) was calculated based on Krausmann (2008), who calculated litter demand per livestock unit (defined by 500 kg life weight) in 1835 for Austrian study regions. We used the lower range of the provided life weight values in order to account for smaller animals in alpine regions. Table S5 provides an overview on the life weights considered in this study. Values were held constant for 1954 and 1973. 
Table S5: Life weights per animal species. Values are taken from Krausmann (2008), considering the lower values of the provided ranges, assuming smaller animals in alpine areas (Krausmann 2008). 
animal 	kg live weight per animal (1865)	
cows	230	
sheep	35	
horses	380	
pigs	60	
goats	45	

The following table (Table S6) provides the gross changes of LULC from 1865 to 2003 (refer to the main text chapter 3). Roughly ¼ of the study area experienced a shift in land use between 1865 and 2003 (Table S6). Forests land and shrublands, followed by extensive grasslands experienced the most significant absolute changes. 24% and 62% of the 2003 forest and shrubland areas were extensive grasslands by 1865. A high share of the extensive grasslands in 1861 was converted to unused grasslands/shrublands which constituted 10% to the land cover in 2003. In contrast, deforestation after 1865 was low, only 7% of the forest areas experienced a shift to another LULC-class, mostly to extensive grasslands as a result of natural hazards, mostly avalanches (expert opinion). Infrastructure areas were mostly build on intensive grasslands and croplands and to a lower degree on forest lands. 

Table S6: Gross changes of LULC in % of the territory between 1865 and 2003. 
             1865 
2003	low productive/
alpine	shrublands	forest land	exstensive grasslands	intensive grasslands/ croplands	Infra-structure	unused grassl.-/ shrublands	Contribution to 2003 area	
low productive
/alpine	21.8%	0.0%	0.0%	0.4%	0.0%	0.0%	0.0%	22%	
shrublands	0.3%	0.8%	0.1%	2.0%	0.0%	0.0%	0.0%	3%	
forest land	0.0%	0.2%	9.2%	3.2%	0.2%	0.0%	0.0%	13%	
exstensive 
grassland	1.4%	0.1%	0.2%	10.7%	0.2%	0.0%	0.0%	13%	
intensive grass./ cropland	0.0%	0.0%	0.1%	0.8%	2.4%	0.0%	0.0%	3%	
infrastructure	0.0%	0.0%	0.1%	0.0%	0.3%	0.0%	0.0%	0%	
unused grass-
/shrublands	0.1%	0.0%	0.2%	9.1%	0.0%	0.0%	0.0%	9%	
Contribution to 1861 area	24%	1%	10%	26%	3%	0%	0%		


2.	Supplementary figures
A
	B
	
Fig. S1 Linear models used to calculate (a) SCpot and (b) NPPpot of potential forest land. The SC and NPP values (blue diamonds) represent the mean values of the upper 20 percentiles of the values in 2003 (refer to chapter 2.4 in the main text), calculated separately for horizontal belts in intervals of 200 m.a.s.l. altitudes. This approach assumes a tendency towards old-growth forests


Fig. S2 Mowing frequency defined as number of mowing times per year (y-axis), depending on altitude (x-axis). The linear model was applied on a digital elevation model for the region of “Innsbruck Land” (provided by the Tyrolean Information System tiris, Land Tirol©). The resulting mowing-frequency map was multiplied with NPPact on areas of intensive grasslands, in order to account for multi-cropping (refer to chapter 2.2 in the main text). Data in the figure are based on results of the project TOP-KLIMA-SCIENCE (SPA/01/2007/133/A/Klimawandel) 


a)	
	
b)	
	
Fig. S3: LULC changes in Neustift from 1865 to 2003. (a) LULC broken down to areas in which LULC was similar in 1865 and 2003 (constant LULC, ca. ¾ of the area) and in which LULC differed (land-use change, ca. ¼ of the area). The latter depicts the underlying land-use process between 1865 and 2003. b) Comparison of average net and gross LULC-changes per year for all time steps broken down to the main LULC classes. 


Fig. S4 HANPPharv in forests broken down to the main harvest types from 1865 to 2003 (refer to chapter 4.3 in the main text).. The secondary axis shows HANPPharv per unit of forest land. Until far into the 20th century the forest system was closely integrated with the livestock system 


3.	References 
Bahn M, Knapp M, Garajova Z, et al (2006) Root respiration in temperate mountain grasslands differing in land use. Global Change Biology 12:995–1006.
Kramer H, Krüger HH (1981) Vorrat und Nutzungsmöglichkeiten forstlicher Biomasse in der Bundesrepublik Deutschland. Der Forst-und Holzwirt 36:33–37.
Krausmann F (2008) Land Use and Socio-economic Metabolism in Pre-industrial Agricultural Systems:  Four  ineteenth-century Austrian Villages in Comparison. 
Leifeld J, Meyer S, Budge K, et al (2015) Turnover of grassland roots in mountain ecosystems revealed by their radiocarbon signature: role of temperature and management. PloS one 10:e0119184.
Patek M (2013) Waldentwicklung und Biomassenveränderung in Neustift im Stubaital in Tirol seit 1834. Diploma thesis, University of Vienna
Rubatscher D, Munk K, Stöhr D, et al (2006) Biomass expansion functions for Larix decidua: a contribution to the estimation of forest carbon stocks. Austrian J For Sci 123:87–101.
Tappeiner U, Tasser E, Leitinger G, et al (2008) Effects of historical and likely future scenarios of land use on above- and belowground vegetation carbon stocks of an alpine valley. Ecosystems 11:1383–1400.
